# Multiple Introductions of *Salmonella* *enterica* Serovar Typhi H58 with Reduced Fluoroquinolone Susceptibility into Chile

**DOI:** 10.3201/eid2611.201676

**Published:** 2020-11

**Authors:** Mailis Maes, Zoe A. Dyson, Ellen E. Higginson, Alda Fernandez, Pamela Araya, Sharon M. Tennant, Stephen Baker, Rosanna Lagos, Myron M. Levine, Juan Carlos Hormazabal, Gordon Dougan

**Affiliations:** University of Cambridge, Cambridge, UK (M. Maes, Z.A. Dyson, E.E. Higginson, S. Baker, G. Dougan);; Monash University, Melbourne, Victoria, Australia (Z.A. Dyson);; London School of Hygiene & Tropical Medicine, London, UK (Z.A. Dyson);; Instituto de Salud Publica de Chile, Santiago, Chile (A. Fernandez, P. Araya, J.C. Hormazabal);; University of Maryland School of Medicine, Baltimore, Maryland, USA (S.M. Tennant, M. M. Levine);; Hospital de Niños de Santiago, Santiago, Chile (R. Lagos)

**Keywords:** South America, Chile, Salmonella Typhi H58, whole-genome sequencing, genotyping, fluoroquinolone resistance, antimicrobial resistance, bacteria, enteric diseases

## Abstract

*Salmonella enterica* serovar Typhi H58, an antimicrobial-resistant lineage, is globally disseminated but has not been reported in Latin America. Genomic analysis revealed 3 independent introductions of *Salmonella* Typhi H58 with reduced fluoroquinolone susceptibility into Chile. Our findings highlight the utility of enhanced genomic surveillance for typhoid fever in this region.

*Salmonella enterica* serovars Typhi, Paratyphi A, and Paratyphi B are the etiologic agents of typhoid and paratyphoid fever. Each year, »11–21 million cases and 128,000–161,000 typhoid-related deaths occur, making typhoid a continued health concern in many low- and middle-income countries, particularly among populations without access to clean water or improved sanitation (*1*). *Salmonella* Typhi H58 lineage, genotype 4.3.1, commonly is associated with multidrug resistance, including resistance to chloramphenicol, ampicillin, and trimethoprim/sulfamethoxazole. In addition, isolates exhibiting resistance to fluoroquinolones have been linked to emergent clades of genotype 4.3.1 in South Asia (*2*), the spread of which could cause major challenges for disease management.

*Salmonella* Typhi H58 4.3.1 is the dominant genotype in many parts of Southeast and South Asia and in East Africa (*3*) and has spread globally but has not been reported in Latin America. Recent data on typhoid fever in South America are limited, and little is known about the population structure and antimicrobial susceptibility profiles of *Salmonella* Typhi on the continent. However, a report of 402 *Salmonella* Typhi isolates collected in Colombia during 2012–2015 showed that only 2.2% were resistant to fluoroquinolones (*4*). In 2016, Colombia reported collecting 204 *Salmonella* Typhi isolates, 12.7% of which exhibited decreased susceptibility to fluoroquinolones (*5*). Because these reports did not include whole-genome sequencing (WGS) data, determining whether isolates were genotype 4.3.1 is not possible.

Before the 1970s, typhoid fever was endemic in parts of South America and hyperendemic in Chile. However, water quality and sanitation improvements across the continent, partly in response to a major cholera epidemic in 1991, likely have contributed to a steep decline in the incidence of typhoid fever (*6*). During 1982–1992, Chile implemented interventions to reduce typhoid fever, including immunizing schoolchildren, prohibiting use of untreated sewage to irrigate crops, and detecting and treating chronic carriers. These interventions drastically reduced transmission and typhoid incidence has declined to 0.2 cases/100,000 persons (*7*), including in the greater Santiago metropolitan region (*8*).

Chile’s epidemiologic surveillance system tracks suspected typhoid fever. Two thirds of cases are confirmed by pathogen isolation from ordinarily sterile body fluids, such as blood or bone marrow. *Salmonella* Typhi isolates from Chile typically are susceptible to antimicrobial agents, but ciprofloxacin resistance has been reported. Among isolates collected during 2009–2016, nearly 2% were ciprofloxacin resistant and 14% displayed intermediate resistance (*9*). We used WGS and bioinformatic analyses to characterize *Salmonella* Typhi isolates from Chile to determine if antimicrobial-resistant H58 4.3.1 isolates have been introduced into South America.

## The Study

**Figure 2:**
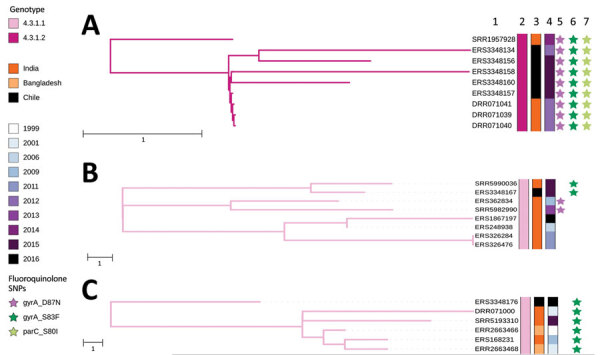
Nearest-neighbor calculations of *Salmonella enterica* serovar Typhi of genotype 4.3.1 and maximum-likelihood phylogenetic trees for 3 introductions of *Salmonella* Typhi genotype 4.3.1 into Chile in the context of their closest *Salmonella* Typhi isolate neighbors. A) Isolate collected during 2012–2014 resembles isolates from South Asia. B) Isolate collected during 2015 resembles isolates from India. C) Isolate collected in 2016 is closely related to a cluster of sequences from India and Bangladesh. Accession numbers, genotypes, countries, and years of isolation are shown. Stars indicate mutations in the quinolone resistance determining region of genes *gyrA*, *gyrA*-S83F and *gyrA-*D87N, and *parC*-S80I. Scale bars indicate SNP distance. SNP, single-nucleotide polymorphism.

We used a HiSeq WGS platform (Illumina, https://www.illumina.com) to generate 150 bp paired-end reads from *Salmonella* Typhi isolates collected during 2011–2017 by Chile’s National Typhoid Surveillance System. We assigned sequences to previously defined genotypes and identified 7 genotype 4.3.1 isolates (Appendix 1). Isolates were obtained from clinical cases in the Santiago metropolitan region: 1 in 2012, 5 in 2015, and 1 in 2016. For global context, we analyzed these 7 genomes and 2,386 publicly available sequences (Appendix 2 Table 1). Among publicly available sequences, 2,326 were genotype 4.3.1 and 60 were non–4.3.1 genotypes (Appendix 1 Table 2). We used the non–4.3.1 genotypes and a *Salmonella* Paratyphi A sequence as an outgroup for phylogenetic tree rooting. We produced clean and filtered SNP alignments (Appendix 1) and used these alignments to infer maximum likelihood phylogenies and specified a generalized time-reversible model and a Gamma distribution to model site-specific rate variation by using GTRGAMMA in RAxML version 8.2.9 (https://github.com/stamatak/standard-RAxML) and 100 bootstrap pseudoreplicates to assess branch support. SNP distances were calculated by using snp-dists (https://github.com/tseemann/snp-dists; Appendix 2). Raw Illumina reads were assembled by using either Velvet version 1.2 (European Bioinformatics Institute, https://www.ebi.ac.uk/~zerbino/velvet) or Unicycler version 0.4.7 (*10*). Assembled reads were input into Pathogenwatch (https://pathogen.watch) to detect nonsynonymous mutations in the quinolone-resistance determining region of *gyrA* and *parC* genes responsible for reduced fluoroquinolone susceptibility. We also used this approach to look for known antimicrobial resistance (AMR) genes. We further screened the sequences from Chile and close genetic relatives (Figure 2) to determine molecular determinants of AMR and known plasmid replicon genes (Appendix 1 Table).

Phylogenomic and SNP analyses confirmed 7 *Salmonella* Typhi genotype 4.3.1 isolates from Chile. Contact tracing implies that 4/5 isolates from 2015 were part of a localized outbreak. We found that the 7 isolates were members of 2 different sublineages, lineage I (4.3.1.1) and lineage II (4.3.1.2), suggesting multiple introductions into Chile. The 2 isolates of lineage I carried a single *gyrA*-S83F mutation predicted to confer reduced susceptibility to fluoroquinolones. The 5 lineage II isolates carried 3 quinolone-resistance determining region mutations, 2 in *gyrA* genes, S83F and D87N, and 1 in *parC*-S80I. Genotype 4.3.1 triple mutants were predicted to be resistant to fluoroquinolones, and isolates of this sublineage with identical mutations have been observed on the subcontinent of India and have been associated with treatment failure (*2*,*11**,**12*). None of the lineage II triple mutants in Chile carried detectable horizontally acquired AMR genes.

**Figure 1:**
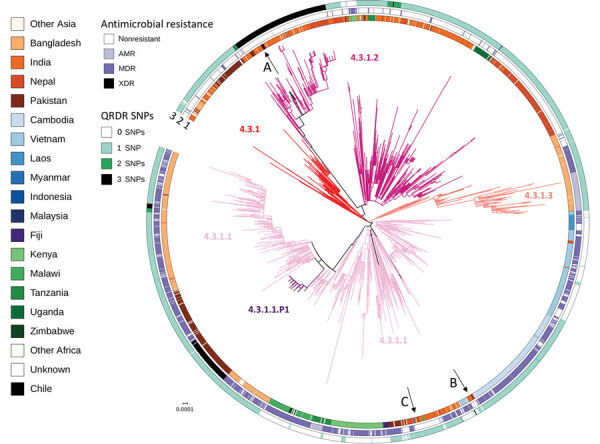
Global context of *Salmonella enterica* serovar Typhi genotype 4.3.1 from Chile. *Salmonella* Typhi H58 genotype 4.3.1-based phylogenetic tree. Branches are colored by genotypes labeled in the tree. A, B, and C arrows indicate isolates from the Chile and the 3 independent introductions. The inner circle indicates country of isolation. The middle circle indicates AMR, excluding reduced susceptibility to fluoroquinolones caused by QRDR SNPs. MDR, including resistance to chloramphenicol, ampicillin, and trimethoprim-sulfamethoxazole; or XDR, multidrug resistance plus resistance to third-generation cephalosporins and reduced susceptibility to fluoroquinolones. The outer circle indicates number of SNPs, 0, 1, 2 or 3, in the quinolone resistance determining region of *gyrA* and *parC* genes. Scale bar indicates nucleotide substitutions per site. AMR, antimicrobial resistance; MDR, multidrug-resistant; QRDR, quinolone-resistance determining region; SNP, single-nucleotide polymorphism; XDR, extremely drug-resistant.

To provide a global contextualization of *Salmonella* Typhi genotype 4.3.1 in Chile, we analyzed the novel sequences alongside 2,326 existing sequences from 31 countries (Figure 1). The 4.3.1.2 triple mutants from Chile formed a closely related phylogenetic cluster (median distance 2 SNPs) with sequences that have the same antimicrobial susceptibility profile isolated from India during 2012–2014, indicating an introduction from South Asia (Figure 2, panel A).

The two 4.3.1.1 isolates from 2015 and 2016 in Chile were in distinct subclades of the tree and were separated by 19 SNPs, suggestive of 2 separate introductions. Of these, 1 introduction was closely related to a 2015 isolate from India (5 SNPs apart) (Figure 2, panel B) and the other was nested in a cluster of sequences from Southeast and South Asia and most closely related (median distance of 20 SNPs) to sequences from India and Bangladesh (Figure 2, panel C).

## Conclusions

Our study confirmed *Salmonella* Typhi H58 genotype 4.3.1 in South America. Phylogenomic and SNP analyses indicate >3 separate genotype introductions into Chile; 5/7 isolates carried 3 distinct mutations, 2 in the *gyrA* gene, at D87N and S83F, and 1 in the *parC* gene at S80I, which are associated with ciprofloxacin resistance. For a high-income country with adequate surveillance, like Chile, the presence of fluoroquinolone-resistant genotype 4.3.1 *Salmonella* Typhi has no immediate implications. However, if this genotype is transferred to low- or middle-income countries in South America, it could have major consequences. Therefore, these data should be of concern to other countries in the region where potential typhoid fever transmission remains high and adequate sanitation might be lacking (*5*,*6*,*10*). Ciprofloxacin is a first-line drug for typhoid fever in much of Latin America, and fluoroquinolone-resistant genotype 4.3.1 would reduce its long-term efficacy.

Most diagnostic laboratories across South America are using pulsed-field gel electrophoresis to study *Salmonella* Typhi epidemiology (*13*), but efforts are underway to implement WGS for epidemiologic surveillance in several countries (*14*,*15*). However, WGS-based approaches for detecting genotype 4.3.1 and understanding trends in genotype population, circulating lineages, and AMR dynamics have not been adopted widely across the region. Our work highlights the need for a uniform WGS platform for global *Salmonella* Typhi monitoring and the need to elucidate the current epidemiology of typhoid fever in South America.

Appendix 1 Additional information on methods of detection for multiple introductions of *Salmonella* Typhi H58 genotype 4.3.1, Chile.

Appendix 2 *Salmonella* Typhi sequences used to investigate multiple introductions of Salmonella Typhi H58 genotype 4.3.1, Chile.
