# Supplementary material for: Multiple Introductions of Salmonella enterica Serovar Typhi H58 with Reduced Fluoroquinolone Susceptibility into Chile
Source: Emerg Infect Dis. 2020 Nov;26(11):2736–40. doi: 10.3201/eid2611.201676 (PMC7588544; doi:10.3201/eid2611.201676)
Supplement: Appendix 1 — Additional information on methods of detection for multiple introductions of Salmonella Typhi H58 genotype 4.3.1, Chile. [file 20-1676-Techapp-s1.pdf]

Article DOI: https://doi.org/10.3201/eid2611.201676

Multiple Introductions of *Salmonella enterica* Serovar Typhi H58 with Reduced Fluoroquinolone Susceptibility, Chile

Appendix 1

Computational Methods

Read Alignment and SNP Analysis

Seven *Salmonella* Typhi isolates collected from Chile during 2012–2016 were identified as being members of genotype 4.3.1, formerly known as H58 under the haplotyping scheme of Roumagnac et al. 2006 (*1*) by using Pathogenwatch (https://pathogen.watch). Two isolates were typed as H58 lineage 1 (genotype 4.3.1.1), and another further 5 were determined to be members of H58 lineage II (genotype 4.3.1.2). Because *Salmonella* Typhi genotype 4.3.1 has not been reported in South America to date, we contextualized these 7 isolates with a global collection of 2,326 previously published genotype 4.3.1 sequenced to date (*2*–*11*; Rahman et al. unpub. data, https://www.biorxiv.org/content/10.1101/664136v1) to determine if these might be recent introductions to the region. Subsequently, *Salmonella* Typhi raw read data from all isolates were mapped to the CT18 reference sequence (accession no. AL513382) by using the RedDog mapping pipeline version 1β.11 (https://github.com/katholt/RedDog). RedDog uses Bowtie version 2.2.9 (*12*) to map reads to the reference genome and SAMtools version 1.3.1 (*13*) to identify SNPs that have phred quality scores >30, and to filter out SNPs supported by <5 reads or with 2.5× the average read depth that represent putative repetitive sequences, or those with ambiguous base calls. For every SNP that passed these criteria in any 1 isolate, the consensus base calls for the SNP locus were extracted from all mapped genomes and those with phred scores <20 were treated as unknown alleles and represented with a gap character. These SNPs were used to confirm the 7 *Salmonella* Typhi genomes from Chile were members of genotype 4.3.1 according to an extended global *Salmonella* Typhi framework (*4*,*14*; Rahman et al. unpub.

data, https://www.biorxiv.org/content/10.1101/664136v1) with the GenoTyphi python script (https://github.com/katholt/genotyphi).

Chromosomal SNPs with confident homozygous allele calls (phred score >20) in all genomes mapped were concatenated to form an alignment of alleles at 15,434 variant sites. SNPs called in both prophage and repetitive sequences of 354 kbp; »74% of bases in the CT18 reference sequence, as defined previously (*2*,*3*; Rahman et al. unpub. data, https://www.biorxiv.org/content/10.1101/664136v1), were excluded along with recombinant regions detected by Gubbins version 2.3.2 (*15*) giving a final alignment length of 11,145 chromosomal SNPs out of a total alignment length of 4,462,203 bp for 2,334 H58 isolates (Appendix 2 Table 1, https://wwwnc.cdc.gov/EID/article/26/11/20-1676-App2.xlsx). SNP alleles from 60 *Salmonella* Typhi genomes, and *Salmonella* Paratyphi A AKU1_12601 (accession no. FM2000053) were included as an outgroup for phylogenetic tree rooting (Appendix 2 Table 2). SNP distances were calculated using snp-dists (https://github.com/tseemann/snp-dists).

Phylogenetic Analysis

Maximum likelihood phylogenetic trees were inferred from the chromosomal SNP alignments with RAxML version 8.2.9 (*16*). A generalized time-reversible model and Gamma distribution were used to model site-specific rate variation (GTR+ Γ substitution model) by using GTRGAMMA in RAxML with 100 bootstrap pseuoreplicates to assess branch support for the maximum likelihood phylogeny. Resulting phylogenies were visualized with Microreact (*17*) (https://microreact.org/project/ktISRBvRz) and iTOL (*18*). Raw read data were deposited in the European Nucleotide Archive and individual accession numbers are listed in Appendix 2 Table 1**.**

Molecular Determination of AMR Genes and Plasmids

Whole sequences where screened using SRST2 version 0.2.0 (*19*) with the ARG-ANNOT (*20*) and PlasmidFinder (*21*) databases to determine molecular determinants of AMR and known plasmid replicon genes

References

1. Roumagnac P, Weill F-X, Dolecek C, Baker S, Brisse S, Chinh NT, et al. Evolutionary history of *Salmonella* Typhi. Science. 2006;314:1301–4. PubMed https://doi.org/10.1126/science.1134933

2. Ingle DJ, Nair S, Hartman H, Ashton PM, Dyson ZA, Day M, et al. Informal genomic surveillance of regional distribution of *Salmonella* Typhi genotypes and antimicrobial resistance via returning travellers. PLoS Negl Trop Dis. 2019;13:e0007620. PubMed https://doi.org/10.1371/journal.pntd.0007620

3. Wong VK, Baker S, Connor TR, Pickard D, Page AJ, Dave J, et al.; International Typhoid Consortium. An extended genotyping framework for *Salmonella enterica* serovar Typhi, the cause of human typhoid. Nat Commun. 2016;7:12827. PubMed https://doi.org/10.1038/ncomms12827

4. Britto CD, Dyson ZA, Duchene S, Carter MJ, Gurung M, Kelly DF, et al. Laboratory and molecular surveillance of paediatric typhoidal *Salmonella* in Nepal: antimicrobial resistance and implications for vaccine policy. PLoS Negl Trop Dis. 2018;12:e0006408. PubMed https://doi.org/10.1371/journal.pntd.0006408

5. Britto CD, Dyson ZA, Mathias S, Bosco A, Dougan G, Jose S, et al. Persistent circulation of a fluoroquinolone-resistant *Salmonella enterica* Typhi clone in the Indian subcontinent. J Antimicrob Chemother. 2020;75:337–41. PubMed https://doi.org/10.1093/jac/dkz435

6. Klemm EJ, Shakoor S, Page AJ, Qamar FN, Judge K, Saeed DK, et al. Emergence of an extensively drug-resistant *Salmonella enterica* serovar typhi clone harboring a promiscuous plasmid encoding resistance to fluoroquinolones and third-generation cephalosporins. MBio. 2018;9:e00105–18. PubMed https://doi.org/10.1128/mBio.00105-18

7. Matono T, Morita M, Yahara K, Lee KI, Izumiya H, Kaku M, et al. Emergence of resistance mutations in *Salmonella enterica* serovar Typhi against fluoroquinolones. Open Forum Infect Dis. 2017;4:ofx230. PubMed https://doi.org/10.1093/ofid/ofx230

8. Park SE, Pham DT, Boinett C, Wong VK, Pak GD, Panzner U, et al. The phylogeography and incidence of multi-drug resistant typhoid fever in sub-Saharan Africa. Nat Commun. 2018;9:5094. PubMed https://doi.org/10.1038/s41467-018-07370-z

9. Tanmoy AM, Westeel E, De Bruyne K, Goris J, Rajoharison A, Sajib MSI, et al. *Salmonella enterica* serovar typhi in Bangladesh: exploration of genomic diversity and antimicrobial resistance. MBio. 2018;9:e02112–8. PubMed https://doi.org/10.1128/mBio.02112-18

10. Pham Thanh D, Thompson CN, Rabaa MA, Sona S, Sopheary S, Kumar V, et al. The molecular and spatial epidemiology of typhoid fever in rural Cambodia. PLoS Negl Trop Dis. 2016;10:e0004785. PubMed https://doi.org/10.1371/journal.pntd.0004785

11. Hooda Y, Sajib MSI, Rahman H, Luby SP, Bondy-Denomy J, Santosham M, et al. Molecular mechanism of azithromycin resistance among typhoidal *Salmonella* strains in Bangladesh identified through passive pediatric surveillance. PLoS Negl Trop Dis. 2019;13:e0007868. PubMed https://doi.org/10.1371/journal.pntd.0007868

12. Langmead B, Salzberg SL. Fast gapped-read alignment with Bowtie 2. Nat Methods. 2012;9:357–9. PubMed https://doi.org/10.1038/nmeth.1923

13. Li H, Handsaker B, Wysoker A, Fennell T, Ruan J, Homer N, et al. 1000 Genome Project Data Processing Subgroup. The sequence alignment/map format and SAMtools. Bioinformatics. 2009;25:2078–9. PubMed https://doi.org/10.1093/bioinformatics/btp352

14. Wong VK, Baker S, Connor TR, Pickard D, Page AJ, Dave J, et al.; International Typhoid Consortium. An extended genotyping framework for *Salmonella enterica* serovar Typhi, the cause of human typhoid. Nat Commun. 2016;7:12827. PubMed https://doi.org/10.1038/ncomms12827

15. Croucher NJ, Page AJ, Connor TR, Delaney AJ, Keane JA, Bentley SD, et al. Rapid phylogenetic analysis of large samples of recombinant bacterial whole genome sequences using Gubbins. Nucleic Acids Res. 2015;43:e15. PubMed https://doi.org/10.1093/nar/gku1196

16. Stamatakis A. RAxML-VI-HPC: maximum likelihood-based phylogenetic analyses with thousands of taxa and mixed models. Bioinformatics. 2006;22:2688–90. PubMed https://doi.org/10.1093/bioinformatics/btl446

17. Argimón S, Abudahab K, Goater RJE, Fedosejev A, Bhai J, Glasner C, et al. Microreact: visualizing and sharing data for genomic epidemiology and phylogeography. Microb Genom. 2016;2:e000093. PubMed https://doi.org/10.1099/mgen.0.000093

18. Letunic I, Bork P. Interactive Tree Of Life (iTOL): an online tool for phylogenetic tree display and annotation. Bioinformatics. 2007;23:127–8. PubMed https://doi.org/10.1093/bioinformatics/btl529

19. Inouye M, Dashnow H, Raven LA, Schultz MB, Pope BJ, Tomita T, et al. SRST2: rapid genomic surveillance for public health and hospital microbiology labs. Genome Med. 2014;6:90. PubMed https://doi.org/10.1186/s13073-014-0090-6

20. Gupta SK, Padmanabhan BR, Diene SM, Lopez-Rojas R, Kempf M, Landraud L, et al. ARG-ANNOT, a new bioinformatic tool to discover antibiotic resistance genes in bacterial genomes.

Antimicrob Agents Chemother. 2014;58:212–20. PubMed https://doi.org/10.1128/AAC.01310-13

21. Carattoli A, Zankari E, García-Fernández A, Voldby Larsen M, Lund O, Villa L, et al. In silico detection and typing of plasmids using PlasmidFinder and plasmid multilocus sequence typing. Antimicrob Agents Chemother. 2014;58:3895–903. PubMed https://doi.org/10.1128/AAC.02412-14

Appendix Table. Metadata and SRST2 results from isolates of *Salmonella* Typhi H58 from Chile and nearest neighbors

Accession no.	Original publication	Genotype	Country	Year	QRDR mutations		Resistance genes	Plasmids
					gyrA	*parC*		
SRR1957928	Ingle et al. 2019 (*2*)	4.3.1.2	India	2014	D87N, S83F	S80I	–	–
ERS3348134	This study	4.3.1.2	Chile	2012	D87N, S83F	S80I	–	–
ERS3348156	This study	4.3.1.2	Chile	2015	D87N, S83F	S80I	–	–
ERS3348160	This study	4.3.1.2	Chile	2015	D87N, S83F	S80I	–	–
ERS3348158	This study	4.3.1.2	Chile	2015	D87N, S83F	S80I	–	–
ERS3348157	This study	4.3.1.2	Chile	2015	D87N, S83F	S80I	–	–
DRR071039	Matono et al. 2017 (*7*)	4.3.1.2	India	2012	D87N, S83F	S80I	–	–
DRR071040	Matono et al. 2017 (*7*)	4.3.1.2	India	2012	D87N, S83F	S80I	–	–
DRR071041	Matono et al. 2017 (*7*)	4.3.1.2	India	2012	D87N, S83F	S80I	–	–
SRR5990036	Ingle et al. 2019 (2)	4.3.1.1	India	2015	S83F	–	–	pHCM2
ERS3348167	This study	4.3.1.1	Chile	2015	S83F	–	–	pHCM2
ERS362834	Wong et al. 2016 (*3*)	4.3.1.1	India	2009	D87N	–	–	pHCM2
SRR5982990	Ingle et al. 2019 (*2*)	4.3.1.1	India	2016	D87N	–	–	pHCM2
ERS1867197	Britto et al. 2020 (*5*)	4.3.1.1	India	2016	–	–	–	pHCM2
ERS248938	Wong et al. 2016 (*3*)	4.3.1.1	India	2006	–	–	–	pHCM2
ERS326284	Wong et al. 2016 (*3*)	4.3.1.1	India	2011	–	–	–	pHCM2
ERS326476	Wong et al. 2016 (*3*)	4.3.1.1	India	2011	–	–	–	pHCM2
ERS3348176	This study	4.3.1.1	Chile	2016	S83F	–	–	–
DRR071000	Matono et al. 2017 (*7*)	4.3.1.1	India	2001	S83F	–	–	–
SRR5193310	Ingle et al. 2019 (*2*)	4.3.1.1	India	2015	S83F	–	–	–
ERR2663466	Tanmoy et al. 2018 (*9*)	4.3.1.1	Bangladesh	1999	S83F	–	–	–
ERS168231	Wong et al. 2016 (*3*)	4.3.1.1	India	2006	S83F	–	–	–
ERR2663468	Tanmoy et al. 2018 (*9*)	4.3.1.1	Bangladesh	2001	S83F	–	–	–

*QDR, quinolone resistance determining region.
